# Anti-inflammatory effects of interleukin-23 receptor cytokine-binding homology region rebalance T cell distribution in rodent collagen-induced arthritis

**DOI:** 10.18632/oncotarget.9309

**Published:** 2016-05-11

**Authors:** Wei Guo, Dongmei Yu, Xin Wang, Cheng Luo, Yucong Chen, Wen Lei, Chen Wang, Yaoyao Ge, Wenyao Xue, Qiqi Tian, Xiangdong Gao, Wenbing Yao

**Affiliations:** ^1^ State Key Laboratory of Natural Medicines, School of Life Science and Technology, China Pharmaceutical University, Nanjing, China

**Keywords:** collagen-induced arthritis, IL-23, Th17, Treg, Th9, Immunology and Microbiology Section, Immune response, Immunity

## Abstract

IL-23 is an important cytokine to regulate Th17 cell differentiation and promote the proliferation of inflammatory cells in Th17-mediated autoimmune diseases. The collagen-induced arthritis (CIA) in rat is a model of rheumatoid arthritis characterized by pronounced inflammatory auto-responses from B and T cells, especially Th17 cells in lesions. In the present study, we used rhIL23R-CHR to block the IL-23 signaling pathway to probe the importance of IL-23 in misbalancing the ratio of Th17/Th9/Treg cells in CIA rats. After treatments with rhIL23R-CHR, the CIA rats showed a significant decrease of secretions of IL-17 and IL-9, whereas FoxP3 was activated in the process, indicating that IL-23 can manipulate the balance of Th17/Th9/Treg cells. Similar to the animal model, IL-23 also possessed remarkable proinflammatory effects on human fibroblast-like synoviocyte cells (HFLS), showing synergetic outcomes with TNF-α. Together, IL-23 could act as a modulator to imbalance the ratio of Th17/Th9/Treg cells, and rhIL23R-CHR could serve as a potential therapeutic agent for RA patients.

## INTRODUCTION

Rheumatoid arthritis (RA) is an autoimmune disease with the deterioration of cartilage and bone destruction characterized by chronic inflammation of the synovial membrane [1]. Currently, RA affects 1% of the adult population worldwide and occurs twice as frequently among women than men, leading to serious reduction of the quality of life [2, 3]. Current treatment options for RA patients include disease modifying antirheumatic drugs, non-steroidal anti-inflammatory drugs and steroid and biological response modifiers [4]. Among them, five monoclonal antibody drugs targeting TNF-α (infliximab, adalimumab, etanercept, certolizumab and golimumab) have been approved for the treatment of RA [5]. Although the antibody drugs have shown remarkable efficacy in RA patients, there are still 30% patients showing tolerance to their treatments [6]. Therefore, there is an urgent need of developing novel therapies with different approaches for RA patients, especially given the strong inflammatory reactions associated with RA process.

In both collagen-induced arthritis (CIA) model and RA patients, T cells are largely involved in RA development, and particularly T helper 17 (Th17) cells are a major player [2]. Meanwhile, it was reported that the density of Th17 cells in non-reacting patients against TNF-α treatment is much higher than those sensitive patients, suggesting that the inflammatory effects of IL-17A in RA development are independent from TNF-α mediation [7]. Th17 cells, characterized by their unique secretion of IL-17, IL-21 and IL-23, are believed to play a central role in promoting osteoclastogenesis in CIA [8–10]. For example, synovial fibroblasts recruited Th17 cells can extensively proliferate and secrete inflammatory cytokines such as IL-17, IL-21 and IL-23, which obviously worsens the disease conditions in the inflamed sites [2]. Within the cytokines secreted by Th17 cells, IL-23 has been demonstrated as a key cytokine in Th17 cell differentiation [11–13]. Knockout of IL-23 gene in mice results in 100% resistance to the induction of experimental autoimmune encephalomyelitis (EAE) and CIA [14]. In addition, the balance of Th17/Treg cells can be changed by the monoclonal antibodies against IL-23, and the direct inhibition of IL-23 can significantly improve the conditions of autoimmune diseases, which further highlights the importance of IL-23 in Th17 and Treg cells [15–18].

In addition to IL-23, Elyaman et al. reported that Th17 cells produce a large amount of IL-9 that acts in an autocrine fashion on both Th17 cells and FoxP3+ nTreg cells [19]. IL-9 is a γc-family cytokine that regulates a range of immune responses, including promoting angiogenesis [14, 20]. On the other hand, in response to TGF-β and IL-4 stimulation from naive CD4 cells, Th9 cells also secret IL-9 and participate in the immune reactions [21]. Ciccia et al. demonstrated that both IL-9 and Th9 cells are over-presented in synovial tissues of RA in correlation with the degree of histological organization of B and T cells in ectopic lymphoid structures [22]. Consequently, Th9 cells with high expression of IL-9, in accordance with Th17 cells, may work together to aggravate the pathological process of RA. Therefore, the blockage of IL-23 signaling pathway primarily responsible for Th17 cell differentiation may also affect Th9 cells to decrease the expression of IL-9 and other pro-inflammatory cytokines.

In patients, synovial cells are known to play a fundamental role in joint damage during RA, although the etiology of RA is not completely understood [23]. HFLS-RA is human fibroblast-like synoviocytes with high proliferating ability and susceptibility isolated from RA patients [24]. IL-23 produced by mononuclear cells in synovial fluid of RA patients can promote inflammatory responses through inducing IL-8 and IL-6 production [25]. Meanwhile, in the presence of TNF-α, HFLS-RA patients typically secrete a large amount of pro-inflammatory cytokines. Thus, HFLS cells can be used to resemble a certain population of RA patients.

Thus far, compelling evidence from animal models and RA patients has suggested that IL-23 could be a key regulator to control the progression and treatment outcomes for RA [26]. With this regard, we postulate that IL-23 may not only affect the balance of Th17/Treg cells, but also regulate Th9 cell population in the pathogenesis of RA, and consequently targeting IL-23 could be a new approach for RA treatment or to overcome the tolerance against TNF-α treatment. We have previously shown that human IL-23 receptor cytokine-binding homology region (rhIL23R-CHR), a truncated extracellular domain of IL23R, is able to directly bind IL-23 for influencing downstream signaling process. This recombinant protein was capable of blocking IL-23/IL-17 pathway to suppress IL-23-mediated production of IL-17a [27, 28]. In this study, to explore the possibility of inhibiting IL-23 to treat RA and/or anti-TNF-α tolerant RA, we use rhIL23R-CHR to evaluate the critical roles of IL-23 in conjunction with TNF-α in CIA model and HFLS cells.

## RESULTS

### rhIL23R-CHR effectively repressed CIA process

**Figure 1:**
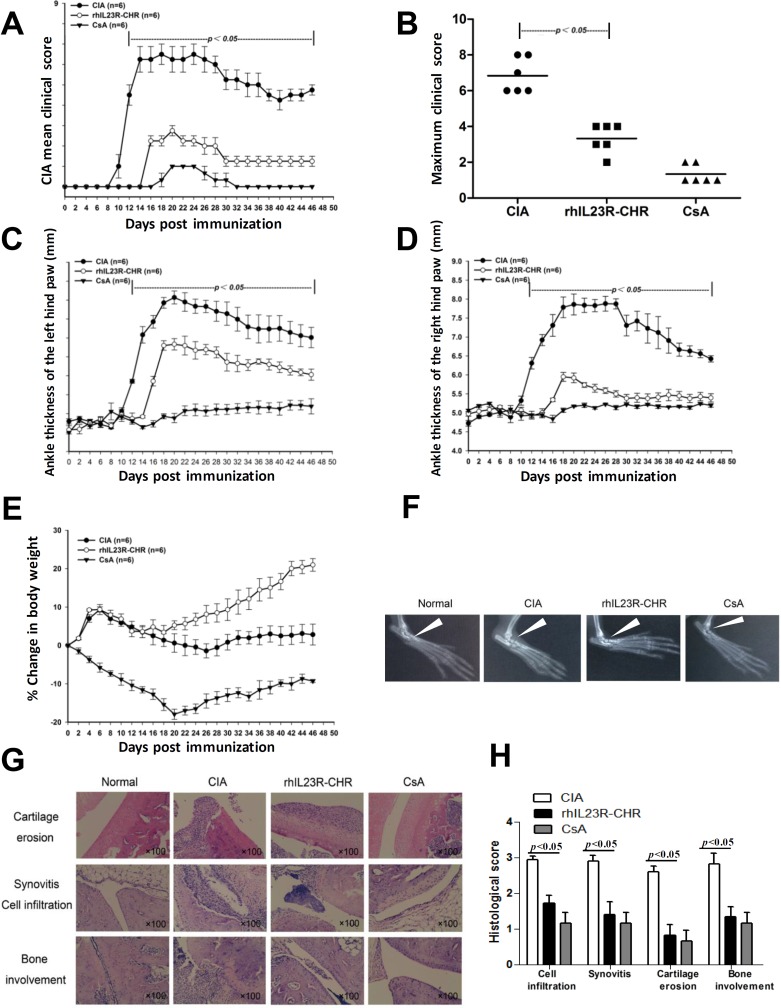
rhIL23R-CHR alleviated the symptoms and delayed disease onset in CIA rats **A.** Mean clinical score of rats treated with rhIL23R-CHR, CsA or vehicle; **B.** Maximum clinical score stood for the peak severity of an individual rat in rhIL23R-CHR, CsA or vehicle-treated group; **C.** Ankle thickness of the left hind paws were measured by vernier caliper; **D.** Ankle thickness of the right hind paws were measured by vernier caliper; **E.** Percent change in body weight starting days post immunization. **F.** Radiographic analysis of joint tissues from each group; **G.** H&E staining of ankle joints obtained from rhIL23R-CHR, CsA or vehicle-treated group; **H.** Pathology scores of cell infiltration, synovitis, cartilage erosion and bone involvement in ankle joints. Data are expressed as mean ± SD. *p* < 0.05; *p* < 0.01; *p* < 0.001.

The treatment effects of rhIL23R-CHR were first investigated in CIA, a widely used animal model of rheumatoid arthritis. Wistar rats were immunized with Chicken type II collagen (CII) in complete freund's adjuvant (CFA) on day 0 and a booster immunization in incomplete freund's adjuvant (IFA) on day 7, followed by administration of rhIL23R-CHR or cyclosporin A (CsA), a classic immunosuppressant as a positive control, on day 0, then re-injected every day. In CIA rats, clinical scores increased progressively from the first onset on day 10. In the meantime, both rhIL23R-CHR and CsA treatments resulted in a delayed disease onset and reduced disease severity based on the calculation of CIA mean clinical score or individual maximum clinical score (Figure 1A, 1B). In addition, ankle swelling was also inhibited by both rhIL23R-CHR and CsA treatments (Figure 1C, 1D). The body weight in CsA treatment group was sharply decreased, due possibly to its non-specific immunosuppression, despite its effectiveness against CIA (Figure 1E). By contrary, blockage of the binding between IL-23 and its receptor by rhIL23R-CHR was also effective to CIA with much less reduction of body weight compared to CsA treatment group (Figure 1E). Radiography of ankle joins was taken to show mild bone destruction in rhIL23R-CHR treatment (Figure 1F). To determine the cause of rhIL23R-CHR on the improvement of arthritis score, ankle joints were removed after sacrificing the rats on day 24 for H&E staining. Histopathological analyses indicated that the rats after rhIL23R-CHR treatment displayed slighter hyperplasia of synovial membrane, fewer inflammatory cells infiltrated to synovium and lower degree of bone involvement compared to the control (Figure 1G, 1H).

### rhIL23R-CHR released the inflammation of CIA rats

**Figure 2:**
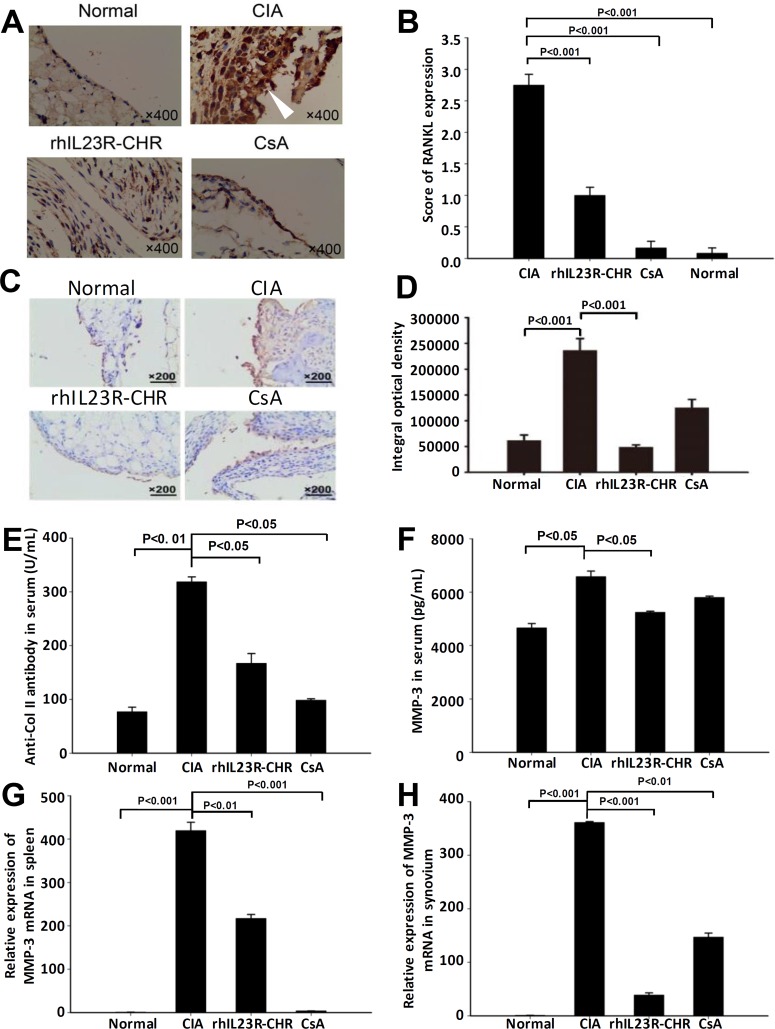
rhIL23R-CHR inhibited the infiltration of inflammatory cells in CIA rats **A.** RANKL immunohistochemistry analysis of swelling paws from rhIL23R-CHR, CsA or vehicle-treated group; **B.** Immunohistochemical scores of RANKL expression; **C.** Immunohistochemistry for VEGF in ankle joints obtained from rhIL23R-CHR, CsA or vehicle-treated group; **D.** The integral optical density of every group; **E.** The level of anti-CII antibody in serum quantified by ELISA; **F.** The level of MMP-3 in serum quantified by ELISA; **G.** The level of MMP-3 mRNA expression in the spleens analyzed by Q-PCR; **H.** MMP-3 mRNA expression in the synovium analyzed by Q-PCR. Data are representative of three independent experiments and expressed as mean ± SD.

To further clarify the potential causes of the effects by rhIL23R-CHR, the expression of RANKL in synovium was examined by immunohistochemistry. As expected, rhIL23R-CHR administration significantly decreased RANKL expression compared to vehicle group (Figure 2A, 2B). Immunohistochemical analysis of VEGF expression revealed that rhIL23R-CHR could markedly decrease the expression of VEGF in ankle joints (Figure 2C, 2D). In addition, the rats treated with rhIL23R-CHR obviously reduced the expression of anti-Col II antibody, confirming the vital roles of autoantibodies in the pathogenesis of autoimmune diseases (Figure 2E). Furthermore, because MMP-3 is a hydrolytic enzyme released by inflamed synovium and contributes to articular cartilage erosion, MMP-3 activity was assayed to show that rhIL23R-CHR treatment markedly decreased the activity of MMP-3 compared to vehicle group (Figure 2F-2H).

### rhIL23R-CHR downregulated the expression of IL-23 and TNF-α in CIA

**Figure 3:**
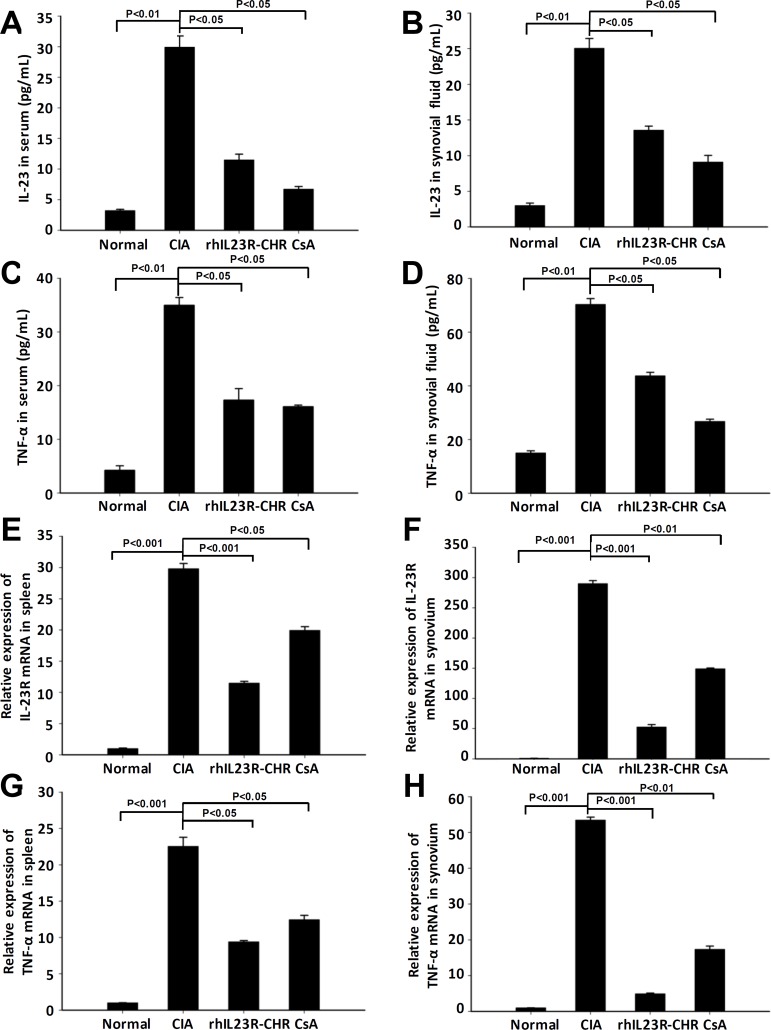
rhIL23R-CHRdown regulated the levels of IL-23, TNF-α in serum and synovial fluid, and IL-23R expression in spleen and synovium **A.**, **B.** The level of IL-23 in serum and synovial fluid quantified by ELISA; **C.**, **D.** The level of TNF-α in serum and synovial fluid determined by ELISA; **E.**, **F.** IL-23R mRNA expression in the spleens and synovium analyzed by Q-PCR; **G.**, **H.** TNF-α mRNA expression in the spleens and synovium analyzed by Q-PCR. Data are representative of three independent experiments and expressed as mean ± SD.

After rhIL23R-CHR administration, the expression of IL-23 and TNF-α in synovial fluid and serum was quantified by ELISA, and their levels were much lower than vehicle group (Figure 3A-3D). Subsequently, Q-PCR analyses indicated that decreased expression of IL-23R and TNF-α was the consequence of reduced transcription by rhIL23R-CHR in the spleen and synovium (Figure 3E-3H). Taken together, these data suggested that rhIL23R-CHR could antagonize IL-23 to suppress the pro-inflammatory functions of TNF-α, IL-23, IL-23R *in vivo*.

### rhIL23R-CHR suppressed IL23 and TNF-α induced inflammatory cytokines responses *in vitro*

**Figure 4:**
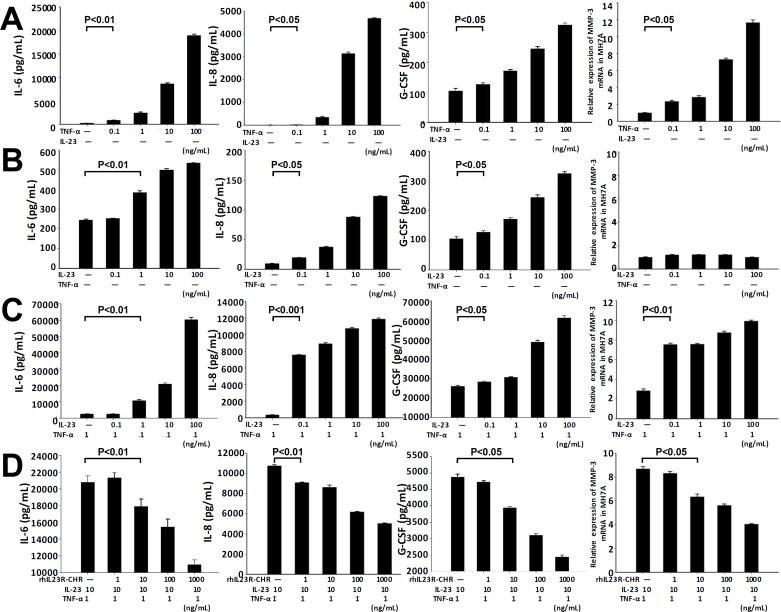
rhIL23R-CHR antagonized the synergistic effects between hIL-23 and hTNF-α on the stimulation of MH7A cells **A.** IL-6, IL-8, G-CSF in the supernatants determined by ELISA and Q-PCR analysis of MMP-3 mRNA expression by TNF-α stimulation; **B.** IL-6, IL-8, G-CSF and MMP-3 by IL-23 stimulation; **C.** IL-6, IL-8, G-CSF in the supernatants determined by ELISA and Q-PCR analysis of MMP-3 mRNA expression by both TNF-α and IL-23 stimulation; **D.** IL-6, IL-8, G-CSF in the supernatants determined by ELISA and Q-PCR analysis of MMP-3 mRNA expression by rhIL23R-CHR treatment. Data are representative of three independent experiments and expressed as mean ± SD.

MH7A is an immortalized cell line of synovial fibroblasts from articular cavity in RA patients. To confirm and justify our findings in CIA rats, MH7A cell line was used to assess the responses by treating with hTNF-α and hIL-23 in this study. Stimulation of MH7A cells with hTNF-α alone at various concentrations resulted in significant increase of IL-6, IL-8, G-CSF and MMP-3 (Figure 4A). However, single treatment by hIL-23 had little effects on the transcription level of MMP-3 although the levels of IL-6, IL-8 and G-CSF were increased (Figure 4B). Interestingly, in the presence of both hIL-23 and hTNF-α, MH7A cells prominently promoted the expression of IL6, IL-8, G-CSF and MMP-3 compared to hTNF-α alone (Figure 4C), and the synergistic effects of hIL-23 and hTNF-α were in a dose-dependent manner. To explore whether rhIL23R-CHR could affect the proinflammatory effects of hIL-23, different concentrations of rhIL23R-CHR were added to the combined treatment with hIL-23 and hTNF-α, and a suppression of the pro-inflammatory effects induced by hIL-23 was observed from rhIL23R-CHR treatment (Figure 4D), suggesting that rhIL23R-CHR could directly neutralize hIL-23 effects in the articular cavity.

### rhIL23R-CHR prevented the development of Th17 population

**Figure 5:**
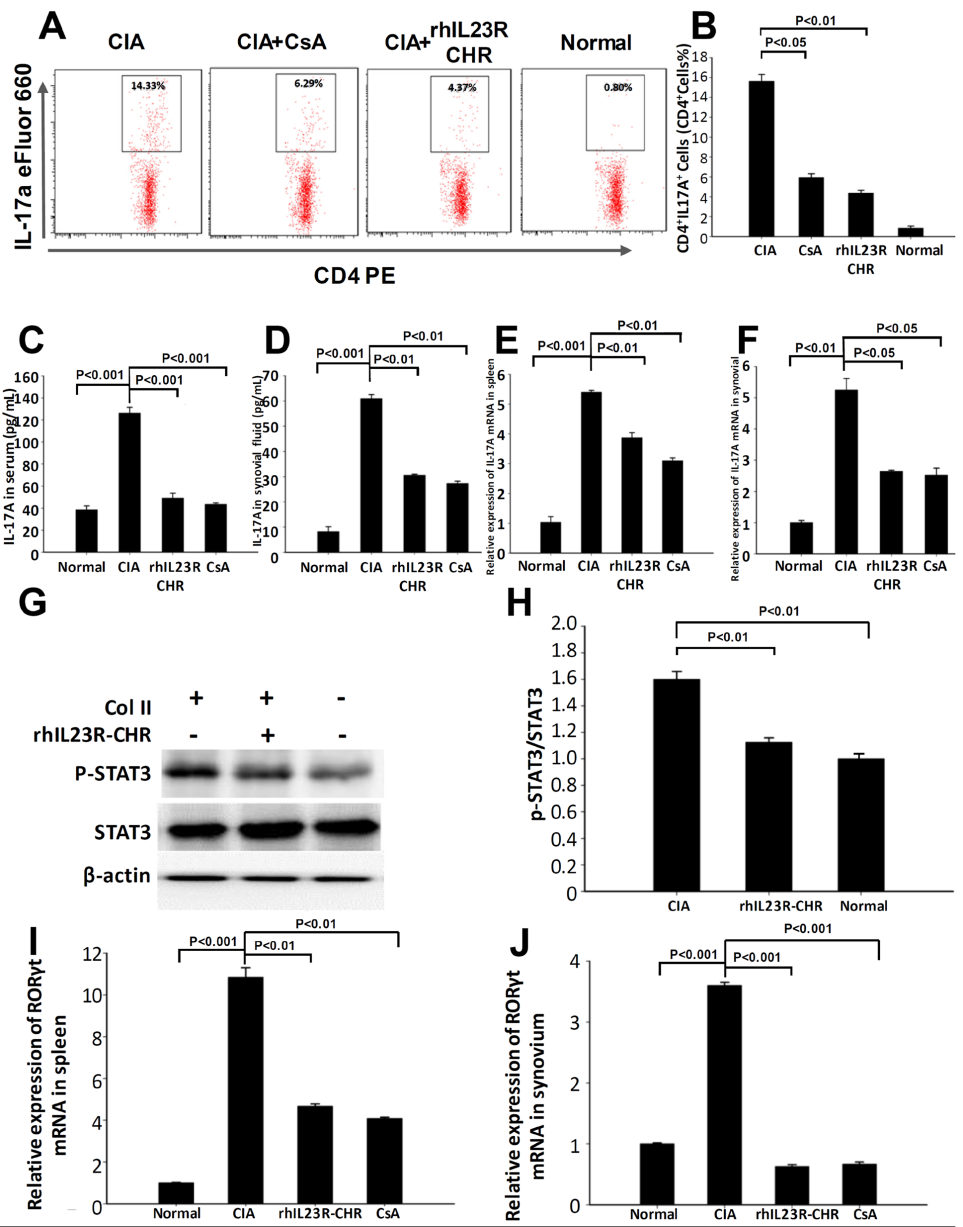
rhIL23R-CHR downregulated Th17 cell differentiation through STAT3/ RORγt **A.** The percentage of Th17 cells in CD4^+^ lymphocyte gate derived from the spleen in treated or control CIA rats analyzed by flow cytometry; **B.** Percentages of cells with positive expression of these antigens in the spleen; **C.**, **D.** The expression level of IL-17A in serum and synovial fluid determined by ELISA; **E.**, **F.** IL-17A mRNA expression in the spleens and synovium analyzed by Q- PCR. **G.** Splenocytes from rhIL23R-CHR-treated rats or control CIA rats analyzed by Western blot; **H.** Gray density analysis of p-STAT3/STAT3 of each group, and the p-STAT3/STAT3 of normal group was normalized to 1; **I.**, **J.** The mRNA abundance of RORγt measured in spleen and synovium. Data are representative of three independent experiments and are expressed as mean ± SD.

Given that IL-23R is a receptor homologous molecule to affect Th17 cell differentiation, the therapeutic effects by rhIL23R-CHR could attribute to the failure of Th17 cell accumulation in the spleens. To examine whether rhIL23R-CHR could inhibit Th17 cell development *in vivo*, the rats were sacrificed on day 24 after first immunization and the splencytes were assayed by FACS. Indeed, the therapeutic effects of rhIL23R-CHR in CIA model were closely associated with the significant decrease of IL-23 concentration in rats (Figure 3A, 3B). Meanwhile, this decrease led to a prominent reduction in the percentage of Th17 cells in the CD4+ subset by the treatment (Figure 5A, 5B), and sera from rhIL23R-CHR-treated rats contained much less IL-17A (Figure 5C-5F), implying that the decrease of Th17 cell population might imbalance the ratio of Th17 and Treg cells. Since phosphorylation of STAT3 is required to induce Th17 cell development by stimulating the key transcriptional factor RORγt, whether rhIL23R-CHR would inhibit STAT3 phosphorylation and downregulate mRNA level of RORγt *in vivo* was subsequently examined. Western blots of lysated splencytes showed that rhIL23R-CHR treatment significantly inhibited STAT3 phosphorylation compared to vehicle (Figure 5G, 5H). In parallel, Q-PCR results also revealed that mRNA level of RORγt was remarkably decreased after rhIL23R-CHR treatment (Figure 5I, 5J).

### rhIL23R-CHR regulated Treg population and function in CIA rats

**Figure 6:**
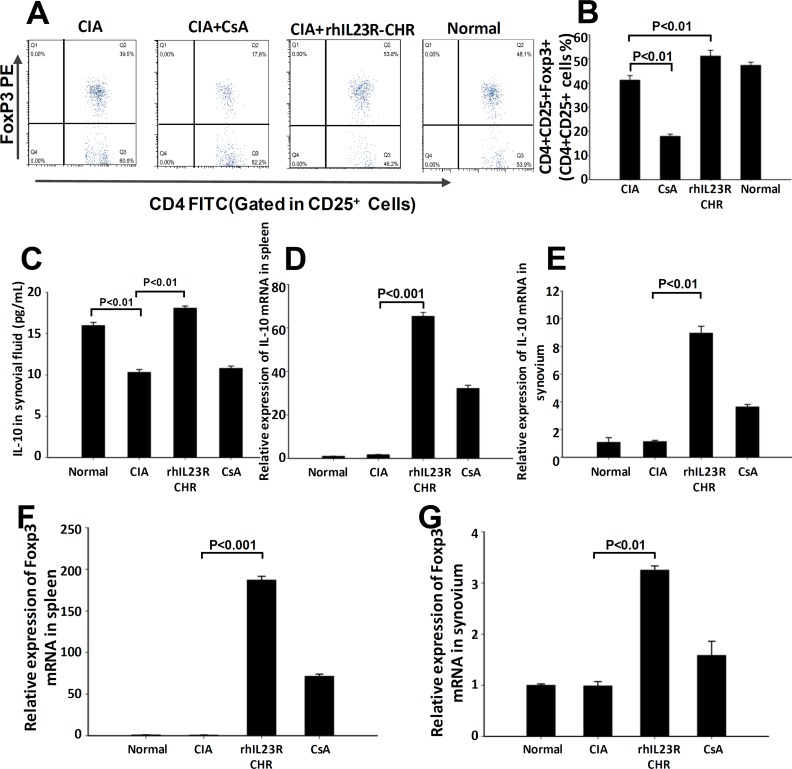
rhIL23R-CHR increased Treg cell differentiation through Foxp3 regulation **A.** The percentage of Treg cells in CD25^+^ lymphocyte gate derived from the spleen in treated or control CIA rats analyzed by flow cytometry; **B.** Percentages of cells with positive expression of these antigens in the spleen; **C.** The expression level of IL-10 determined by ELISA in synovial fluid; **D.**, **E.** IL-10 mRNA expression in the spleensand synovium from CIA rats analyzed by Q-PCR; **F.**, **G.** Foxp3 mRNA in the spleen and synovium analyzed by Q-PCR. Data are representative of three independent experiments and are expressed as mean ± SD.

Given the fact that rhIL23R-CHR could prevent against Th17 cell development in CIA rats, whether this effect makes a difference in the differentiation of Treg cells was an important issue to be addressed. First, splenocytes from every rat in different groups were acquired and stained with PE-Foxp3, FITC-CD4 and APC-CD25 antibodies for FACS assay. The assay results indicated that rhIL23R-CHR treatment could upregulate the differentiation of Treg cells compared to vehicle (Figure 6A, 6B). In addition, rhIL23R-CHR was able to significantly increase the level of IL-10 as determined by ELISA and Q-PCR (Figure 6C-6E). Next, Foxp3, the signature transcriptional factor for Treg cells, was quantified by Q-PCR. Compared to vehicle, the mRNA level of FoxP3 in rhIL23R-CHR-treated rats was significantly upregulated (Figure 6F, 6G).

### rhIL23R-CHR reduced Th9 population in CIA rats

**Figure 7:**
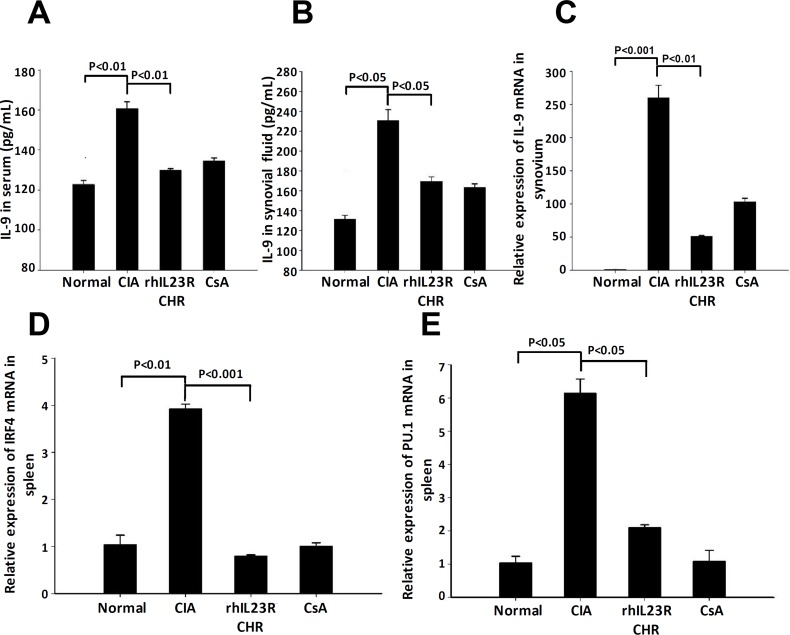
rhIL23R-CHR reduced the Th9 cells through IRF4/PU.1 regulation **A.**, **B.** The expression level of IL-9 in serum and synovial fluid determined by ELISA; **C.** IL-9 mRNA expression in the synovium from CIA rats analyzed by Q-PCR; **D.**, **E.** IRF4 and PU.1in the spleen and synovium determined by Q-PCR. Data are representative of three independent experiments and are expressed as mean ± SD.

Since Th9 cells have been suggested to involve in the progression of CIA, we then examined the level of IL-9, a signature cytokine of Th9 cells. Surprisingly, ELISA and Q-PCR analyses indicated that IL-9 was significantly lower in serum and synovial fluid (Figure 7A-7C), suggesting that the population of Th9 cells was changed as well. At the transcription level, other two Th9-associated transcriptional factors, IRF4 and PU.1, were significantly down-regulated by the treatment of rhIL23R-CHR (Figure 7D, 7E), further confirming that the decrease of Th9 cell population was regulated by their transcription factors.

## DISCUSSION

Currently, immunosuppressive agents are used as alternative therapy for the treatment of autoimmune diseases [4]. However, they often exhibit severe adverse effects, such as nephrotoxicity, cardiotoxicity, hypertension and hepatotoxicity, due mainly to the lack of specificity [29]. In our previous work, rhIL23R-CHR, a homologous protein of the endogenous extracellular receptor domain, could be used as a natural blocker against IL-23 [28], and the prokaryotically expressed human IL23R-CHR protein could inhibit the development of mouse and human Th17 cells *in vitro* [27]. In the present study, we intended to investigate the antagonistic effects of rhIL23R-CHR and probe its mechanism of action in CIA rat model. CIA is the most commonly used animal model for RA, sharing the same pathologic features with RA patients [30]. The homolog of IL23R-CHR genes between human and rat is 82.98%, this human sequence-based recombinant protein could be utilized as a molecular probe to exploit the relationship between the blockage of IL-23 signaling pathway and Th17 /Treg/Th9 cell balance in the rat model. Previous results indicated that knockout mice lacking the expression of IL-23 or IL23 receptor results in suppressed development of CIA [31, 32]. Similarly, we found in this study that rhIL23R-CHR could achieve the same results as the gene knockout, and both rhIL23R-CHR and CsA treatments prevented disease progression and ameliorated disease severity. Because of the non-specific immunosuppressive nature of CsA, we paid special attention to the differences between rhIL23R-CHR and CsA on T cell function and distribution in the pathological process of RA in this study to differentiate the specificity and rationalize the potential benefits of targeting a specific signaling pathway. After CsA treatment, T cells (Th17/Treg/Th9) differentiation were completely inhibited, where as rhIL23R-CHR treatment resulted in a reduced differentiation of Th17/Th9 cells and an increased differentiation of Treg cells in CIA rats. Since Treg cells are well recognized as regulatory cells to inhibit the incidence of autoimmune diseases [33], the increase of Treg differentiation by rhIL23R-CHR treatment was in a good agreement with its therapeutic effects shown in CIA model. In addition, when body weight was concerned, the remarkable loss by CsA treatment vs. unchanged body weight by rhIL23R-CHR treatment certainly was an obvious indicator for the advantages of rhIL23R-CHR. These results strongly indicated that rhIL23R-CHR could be an alternative therapy for autoimmune diseases, which may possess a variety of benefits compared to classical immunosuppressive agents.

From pathological point of view, TNF-α, the key cytokine in the induction of CIA model, has been a therapeutic target for monoclonal antibodies or receptor fusion blockers [34]. Although a great success has been achieved in the design and development of anti-TNF-α-based therapeutics, TNF-α gene deletion in mice have still shown 20% probability of developing into autoimmune diseases in the animal models. In patients, due to individual differences and other unknown factors, those who poorly responded to anti-TNF-α therapy are urgently in the need of alternative therapies to overcome the tolerance [35]. Given the fact that knockout of IL-23 gene in mice exhibited 100% resistance to EAE and CIA induction [14], IL-23 and its associated signaling pathway could be promising therapeutic targets. In the present study, high levels of both TNF-α and IL-23 in serum and synovial fluid were detected in CIA model. However, after rhIL23R-CHR treatment, the accumulation of IL-23 and TNF-α was remarkably lowered. To further justify the potential roles of IL-23 in RA patients, HFLS-RA, an immortal cell line of human fibroblasts from knee joints of RA patients, was utilized to assess the regulatory effects of IL-23 on other cytokines and pro-inflammatory factors. We found that stimulation by TNF-α alone could upregulate the expression of IL-6, IL-8, G-CSF and MMP-3 in this cell line. When the cells were co-stimulated by IL-23 and TNF-α, the pro-inflammatory activity was significantly enhanced. As expected, addition of rhIL23R-CHR markedly inhibited the stimulatory effects by both IL-23 and TNF-α. The results in CIA rats and HFLS-RA cell line obviously indicated that IL-23 solely participates in RA process or synergizes with TNF-α to exert its role as a strong inflammatory cytokine. Therefore, blockage of IL-23-mediated signaling pathway can represent a therapeutic approach for the treatment of RA or anti-TNF-α tolerant RA patients, and rhIL23R-CHR could be served as a therapeutic agent for this purpose. The present results warrants that single treatment by rhIL23R-CHR or combination treatment with TNF-α blockers can be assessed and compared in clinical settings.

Apart from high expression of the pro-inflammatory cytokines, angiogenesis is another critical event by enhancing the formation or maintenance of pannus and promoting inflammatory cells infiltration in histological section of CIA model [4]. The observation of vascular proliferation in ankle joints of CIA rats in the present study led us to speculate that IL-9 could be involved in the pathogenesis of CIA. To verify this hypothesis, we examined IL-9 level in serum and synovial fluid, and high level of IL-9 production was indeed found. Again, blockage of IL-23 by rhIL23R-CHR showed a significant decrease of IL-9 in serum and synovial fluid. Meanwhile, histological analyses also revealed lower degree of angiogenesis and VEGF expression in ankle joints of CIA rats. Therefore, our results suggested that IL-23 promotes the expression of IL-9, the signature cytokine of Th9 cells [21], thereby expediting the growth of blood vessels to increase the severity of the disease.

In conclusion, we have confirmed that IL-23 promotes Th17 cell differentiation via RORγt/STAT3 signaling pathway to upregulate the expression of IL-17A, and increased Th17 cell differentiation further advances the inflammatory disorders for osteoclastogenesis. In addition, IL-23 participated in the differentiation of Th9 cells through upregulating PU.1 and IRF4, and subsequently increased the expression of IL-9 and VEGF to promote the formation of blood vessels and pannus. Moreover, Treg development and the subsequent IL-10 secretion were inhibited by IL-23 to result in the deficiency of immunoregulatory function. The present results from CIA model and the treatment by rhIL23R-CHR clearly indicated that IL-23 can synergize TNF-α to exert stronger pro-inflammatory activity. Given the increasingly important roles of IL-23 in the pathogenesis of RA, modulation of the distribution of Th17/Treg/Th9 cells by rhIL23R-CHR could inhibit or reverse the pro-inflammatory effects in RA progression. Collectively, the present study strongly suggested that rhIL23R-CHR has the potential to serve as a new therapeutic agent for RA patients and/or a complementary therapy in anti-TNF-α tolerant patients.

## MATERIALS AND METHODS

### Animals

Female Wistar rats (6-8 weeks) weighed 160-180 g were purchased from Comparative Medicine Center of Yangzhou University, China. The rats were housed under Specific Pathogen Free conditions and were fed with sterilized food, bedding and water. All animal experimental procedures were conducted in accordance with the Guide for Care and Use of Laboratory Animals as adopted and promulgated by the United States National Institutes of Health, and were approved by Jiangsu Provincial Experimental Animal Manage Committee under Contract scxk (su) 2012-2014, China.

### Generation of rhIL23R-CHR

Purified rhIL23R-CHR used in this study was prepared based on our previous report [28]. The recombinant protein had a purity of 99%. All proteins were treated with Endotoxin affinity Resin (Genscript, USA) to remove the endotoxin before followed assays, the purified proteins were shown to have negligible endotoxin contamination (< 10 EU/mg) by an LAL Chromogenic endotoxin quantization assay(Genscript, USA).

### Collagen-induced arthritis induction and treatment by rhIL23R-CHR

Type II chicken collagen (Sigma, USA) was dissolved in 0.1 M acetic acid at 4 mg/mL overnight at 4°C under sterile condition. Each rat was immunized by intradermally injection of 100 μg CII emulsified in CFA (Sigma, USA) at the base of the tail on day 0. A booster injection was conducted 7 days after the primary immunization with another 100 μg CII emulsified in IFA (Sigma, USA). To evaluate the influence of rhIL23R-CHR on the treatment of CIA model, rats were treated with intravenous administration of rhIL23R-CHR at 1 mg/kg every two days from day 0 to day 20. CsA was given by intragastric administration at 1.5 mg/kg as a positive control. Normal and CIA rats were administrated with an equal volume of PBS at the same time. The severity of the arthritis was scored every two days. Inflammation of two hind paws were graded from 0 to 4: grade 0, paws with no swelling and focal redness; grade 1, paws with swelling of finger joints; grade 2, paws with mild swelling of ankle or wrist joints; grade 3, paws with severe inflammation of the entire paws; and grade 4, paws with deformity or ankylosis. Each paw was graded and the two scores were combined so that the maximum possible score per rat was 8. Clinical scores were based on the sum of two hind paws. In addition, body weight and the diameter of ankle thickness were recorded.

### Immunohistochemical analysis

Rats were euthanized with CO_2_ at the end of treatments on day 24. The hind paws were removed, fixed with neutral buffered 10% formalin, then decalcified for 10 days with 15% EDTA-2Na, embedded in paraffin and sectioned. The paraffin sections were stained for H&E, RANKL(Thermo, USA)and VEGF(Santa cruz, USA). Sections were evaluated blindly by authoritative pathologists. Briefly, inflammation was scored as follows: 0, none; 1, a few inflammatory cells; 2, organization of perivascular infiltrates; and 3, increasing severity of perivascular cuffing with extension into the adjacent tissue. The immunohistochemical of VEGF was analyzed by ImagePro plus6.0 software.

### Radiography

Rats were anesthetized with 10% chloral hydrate, and the X-ray radiography was performed on the 20th day since the first immunization.

### ELISA

Cytokines were measured by commercially available kits as follows:ratIL-23(Cusabio, China), ratIL-17A(Dakewe, China), ratIL- (Dakewe, China), ratIL-10(Dakewe, China), ratMMP-3(Cusabio, China), ratTNF-α(Dakewe, China), and rat anti-Col II antibody(Cusabio, China), humanIL-6(Dakewe, China), humanIL-8(Dakewe, China) and humanGM-CSF(Dakewe, China) were measured by individual ELISA kits according to the instructions of different manufacturers.

### Flow cytometry

The spleens were dissected from the rats, and pass through a sterilized 70 μm nylon cell stainer (BD bioscience, USA) to obtain single cell suspensions in IMDM (containing 10% FBS, Gbico, USA) medium. Red blood cells were lysed with RBC lysis buffer (eBioscience, USA). After stimulated with 2μL/mL Leukocyte Activation Cocktail, with GolgiPlug(BD, USA) for 4-6h, cells were collected and washed, then incubated with anti-rat CD16/CD32 (BD, USA) for 20 min to block non-specific Fc interactions. Then the cells were stained with PE-anti-rat CD4(eBioscience, USA) for 30 min at 4°C in dark. After washing twice with staining buffer (BD, USA),the cells were fixed and permeabilized using Cytofix/Cytoperm solution(BD, USA) for 20min at 4°C in dark,and the intracellular cytokine was stained using eFluor660-anti rat IL-17A (eBioscience, USA) for 30min at room temperature in dark. In addition, for Treg cells, FITC anti-rat CD4(eBioscience, USA), APC anti-rat CD25 (eBioscience, USA)and PE anti-rat Foxp3 (eBioscience, USA) were used for staining. All protocols were per the manufacturer's instructions. FACS analysis was performed on a Becton-Dickinson FACSCalibur(BD, USA) and the data were analyzed through Flowjo software (Tree Star Int,USA).

### Western blotting

Proteins were extracted with RIPA lysis buffer (Beyotime, China) containing 1 mM PMSF (Beyotime, China) and 1 mM phosphatase inhibitor (Pierce, USA). The total proteins were quantified with BCA protein assay kit (Beyotime, China) and readjust to the same concentration before loading to 10% SDS-PAGE. Western blotting was performed by transferring the proteins onto polyvinylidene difluride membranes (Milipore, Germany) using a TransBlot system(Bio-Rad, USA). The membranes were washed in ddH_2_O and then blocked with 5% milk in Tris-buffered saline supplemented with 0.1% Tween 20 (TBST) for 2h at room temperature. After that, the membranes were incubated overnight at 4°C with antibodies at a dilute of 1:1000 against phosphorylated STAT3(Cell Signaling, USA), STAT3(Cell Signaling, USA) and β-actin (Santa Cruz, USA) followed by the incubation with HRP-conjugated secondary antibodies for another 1h at room temperature. The signals were detected using West PicoChemiluminescent Substrate (Pierce, USA) according to manufaturer's instructions.

### Quantitative real-time PCR

The gene expression were detected through quantitative real-time PCR using pre-designed primers by the comparative method of relative quantitation (ΔΔCt). Total RNA was extracted using Trizol Regents (Invitrogen, USA) and the first-strand cDNA was synthesized using TransScript First-Strand cDNA Synthesis SuperMix(Transgen Biotech, China). The quantitative real-time PCR was performed on a ABI Step One Plus Instrument(Applied biosystem, USA) using SYBRgreen Master Mix(Applied biosystem, USA) under standard thermocycler condition. Ratβ-actin and humanβ-actin were used as the internal control for sample normalization. Sequences of PCR primer pairs were summarized in Supplementary Tables 1 and 2.

### Cells and cell culture procedures

MH7A, an immortalized cell line from human primary synoviocyte-like fibroblasts derived from RA patients, were obtained from Jiniou. Inc (Guangzhou, China) at early passage and cultured in DMEM (Gibco, USA) with 10% FBS (Gibco, USA). Cells between passages four and eight were normally used. In brief, 2×10^4^HFLS cells/well were pre-cultured in 24-well plate at 37°C, 5%CO_2_for two days before cytokines (R&D, USA) (and rhIL23R-CHR) were added. IL-6, IL-8, G-CSF in cell supernatants were measured by individual ELISA kits according to manufacturers' instructions. MMP-3 in cell lysates was measured by Q-PCR.

### Statistical analysis

Results are analyzed as the mean±SD. All data represent at least three repeats of independent experiments. The unpaired t test or one way ANOVA with Dunnett's multiple comparison test was used to test statistical significance and a P values of < 0.05 was considered statistically significant.

## SUPPLEMENTARY MATERIAL TABLES



## References

[1] Ceeraz S, Nowak EC, Burns CM, Noelle RJ (2014). Immune checkpoint receptors in regulating immune reactivity in rheumatic disease. Arthritis Research & Therapy.

[2] Komatsu N, Takayanagi H (2012). Inflammation, bone destruction in arthritis: synergistic activity of immune, mesenchymal cells in joints. Frontiers in Immunology.

[3] Cho JH, Feldman M (2015). Heterogeneity of autoimmune diseases: pathophysiologic insights from genetics, implications for new therapies. Nature Medicine.

[4] Kong X, Zhang Y, Liu C, Guo W, Li X, Su X, Wan H, Sun Y, Lin N (2013). Anti-angiogenic effect of triptolide in rheumatoid arthritis by targeting angiogenic cascade. PLoS One.

[5] Avci AB, Feist E, Burmester GR (2015). Biologicals in rheumatoid arthritis: current, future. Rheumatic Musculoskeletal Diseases Open.

[6] Biggioggero M, Favalli EG (2014). Ten-year drug survival of anti-TNF agents in the treatment of inflammatory arthritides. Drug development research.

[7] Andersson KM, Cavallini NF, Hu D, Brisslert M, Cialic R, Valadi H, Erlandsson MC, Silfversward S, Pullerits R, Kuchroo VK, Weiner HL, Bokarewa MI (2015). Pathogenic Transdifferentiation of Th17 Cells Contribute to Perpetuation of Rheumatoid Arthritis during Anti-TNF Treatment. Molecular Medicine.

[8] Ivanov II, McKenzie BS, Zhou L, Tadokoro CE, Lepelley A, Lafaille JJ, Cua DJ, Littman DR (2006). The orphan nuclear receptor RORgammat directs the differentiation program of proinflammatory IL-17+ T helper cells. Cell.

[9] Schmidt-Weber CB, Akdis M, Akdis CA (2007). TH17 cells in the big picture of immunology. The Journal of Allergy and Clinical Immunology.

[10] Chen GJ, Zhang X, Li RS, Fang L, Niu XY, Zheng YX, He DY, Xu R, Zhang JW (2010). Role of Osteopontin in Synovial Th17 Differentiation in Rheumatoid Arthritis. Arthritis & Rheumatism.

[11] Éric T (2012). The IL23/Th17 Pathway as a Therapeutic Target in Chronic Inflammatory Diseases. Inflammation & Allergy - Drug Targets.

[12] Zhou L, Littman DR (2009). Transcriptional regulatory networks in Th17 cell differentiation. Current Opinion in Immunology.

[13] Guo W, Wang C, Wang X, Luo C, Yu DM, Wang YH, Chen YC, Lei W, Gao XD, Yao WB (2015). A novel human truncated IL12rβ1-Fc fusion protein ameliorates experimental autoimmune encephalomyelitis via specific binding of p40 to inhibit Th1, Th17 cell differentiation. Oncotarget.

[14] Furuzawa-Carballeda J, Vargas-Rojas MI, Cabral AR (2007). Autoimmune inflammation from the Th17 perspective. Autoimmunity Reviews.

[15] Tang C, Chen S, Qian H, Huang W (2012). Interleukin-23: as a drug target for autoimmune inflammatory diseases. Immunology.

[16] Noack M, Miossec P (2014). Th17 and regulatory T cell balance in autoimmune and inflammatory diseases. Autoimmunity Reviews.

[17] Benson JM, Peritt D, Scallon BJ, Heavner GA, Shealy DJ, Giles-Komar JM, Mascelli MA (2011). Discovery, mechanism of ustekinumab: a human monoclonal antibody targeting interleukin-12 and interleukin-23 for treatment of immune-mediated disorders. MAbs.

[18] Ju JH, Heo YJ, Cho ML, Jhun JY, Park JS, Lee SY, Oh HJ, Moon SJ, Kwok SK, Park KS, Park SH, Kim HY (2012). Modulation of STAT-3 in Rheumatoid Synovial T Cells Suppresses Th17 Differentiation and Increases the Proportion of Treg Cells. Arthritis & Rheumatism.

[19] Elyaman W, Bradshaw EM, Uyttenhove C, Dardalhon V, Awasthi A, Imitola J, Bettelli E, Oukka M, van Snick J, Renauld JC, Kuchroo VK, Khoury SJ (2009). IL-9 induces differentiation of TH17 cells and enhances function of FoxP3+ natural regulatory T cells. Proceedings of the National Academy of Science of the United States of America.

[20] Rochman Y, Spolski R, Leonard WJ (2009). New insights into the regulation of T cells by gamma(c) family cytokines. Nature Reviews Immunology.

[21] Schmitt E, Klein M, Bopp T (2014). Th9 cells, new players in adaptive immunity. Trends in Immunology.

[22] Ciccia F, Guggino G, Rizzo A, Manzo A, Vitolo B, La Manna MP, Giardina G, Sireci G, Dieli F, Montecucco CM, Alessandro R, Triolo G (2015). Potential involvement of IL-9and Th9 cells in the pathogenesis of rheumatoid arthritis. Rheumatology.

[23] Chen L, Wei XQ, Evans B, Jiang W, Aeschlimann D (2008). IL-23 promotes osteoclast formation by up-regulation of receptor activator of NF-kappaB (RANK) expression in myeloid precursor cells. European Journal of Immunology.

[24] Bartok B, Firestein GS (2010). Fibroblast-like synoviocytes: key effector cells in rheumatoid arthritis. Immunological Reviews.

[25] Liu FL, Chen CH, Chu SJ, Chen JH, Lai JH, Sytwu HK, Chang DM (2007). Interleukin (IL)-23 p19 expression induced by IL-1beta in human fibroblast-like synoviocytes with rheumatoid arthritis via active nuclear factor-kappaB, AP-1 dependent pathway. Rheumatology.

[26] Teng MW, Bowman EP, McElwee JJ, Smyth MJ, Casanova JL, Cooper AM, Cua DJ (2015). IL-12and IL-23 cytokines: from discovery to targeted therapies for immune-mediated inflammatory diseases. Nature Medicine.

[27] Guo W, Luo C, Wang C, Wang YH, Wang X, Gao XD, Yao WB (2014). Suppression of human and mouse Th17 differentiation, autoimmunity by an endogenous Interleukin 23 receptor cytokine-binding homology region. The International Journal of Biochemistry & Cell Biology.

[28] Guo W, Luo C, Wang C, Zhu Y, Wang X, Gao XD, Yao WB (2012). Protection against Th17 cells differentiation by an interleukin-23 receptor cytokine-binding homology region. PLoS One.

[29] Florio S, Ciarcia R, Crispino L, Pagnini U, Ruocco A, Kumar C, D'Andrilli G, Russo F (2003). Hydrocortisone has a protective effect on CyclosporinA-induced cardiotoxicity. Journal of cellular physiology.

[30] Brand DD, Latham KA, Rosloniec EF (2007). Collagen-induced arthritis. Nature Protocols.

[31] Duvallet E, Semerano L, Assier E, Falgarone G, Boissier MC (2011). Interleukin-23: a key cytokine in inflammatory diseases. Annals of Medicine.

[32] Cornelissen F, Asmawidjaja PS, Mus AM, Corneth O, Kikly K, Lubberts E (2013). IL-23 Dependent and Independent Stages of Experimental Arthritis: No Clinical Effect of Therapeutic IL-23p19 Inhibition in Collagen-induced Arthritis. PLoS One.

[33] Feng Y, van der Veeken J, Shugay M, Putintseva EV, Osmanbeyoglu HU, Dikiy S, Hoyos BE, Moltedo B, Hemmers S, Treuting P, Leslie CS, Chudakov DM, Rudensky AY (2015). A mechanism for expansion of regulatory T-cell repertoire and its role in self-tolerance. Nature.

[34] Joosten LA, Helsen MM, van de Loo FA, vanden Berg WB (2008). Anticytokine treatment of established type II collagen-induced arthritis in DBA/1 mice: a comparative study using anti-TNFalpha, anti-IL-1alpha/beta andIL-1Ra. Arthritis & Rheumatism.

[35] Scheinfeld NA (2004). Comprehensive review andevaluation of the side effects of the tumor necrosis factor alpha blockers etanercept, infliximab andadalimumab. Journal of Dermatological Treatment.

