# Supplementary material for: Anti-inflammatory effects of interleukin-23 receptor cytokine-binding homology region rebalance T cell distribution in rodent collagen-induced arthritis
Source: Oncotarget. 2016 May 11;7(22):31800–13. doi: 10.18632/oncotarget.9309 (PMC5077977; doi:10.18632/oncotarget.9309)
Supplement: Supplementary file 1 [file oncotarget-07-31800-s001.pdf]

Anti-inflammatory effects of Interleukin-23 receptor cytokine-binding homology region rebalance T cell distribution in rodent collagen-induced arthritis

Supplementary Material

Suppl. Table 1: RT-PCR primers for rats

Primer name	Sequences
Rat β-actin (Forward)	5’-CCCGCGAGTACAACCTTCT-3’
Rat β-actin (Reverse)	5’-CGTCATCCATGGCGAACT-3’
Rat IL-17A (Forward)	5’-CTTCACCCTGGACTCTGAGC-3’
Rat IL-17A (Reverse)	5’-CCTCAGCGTTGACACAGC-3’
Rat IL-22 (Forward)	5’-CTACACTCCCACCGTTGATG-3’
Rat IL-22 (Reverse)	5’-CCTCCCTTACCAAAGAGCTG-3’
Rat RORγt (Forward)	5’-TACACGGCCTTGGTTCTCAT-3’
Rat RORγt (Reverse)	5’-GCAGATGCTCCACTCTCCTC-3’
Rat IL-23R (Forward)	5’-TGCAAAATGACTCACCCAGT-3’
Rat IL-23R (Reverse)	5’-CAAATTCATCAGGCAGCAGA-3’
Rat TNF-α (Forward)	5’-CCCTGGTACTAACTCCCAGAAA-3’
Rat TNF-α (Reverse)	5’-TGTATGAGAGGGACGGAACC-3’
Rat MMP-3 (Forward)	5’-GATGTAGAGGGCCTAACCCA-3’
Rat MMP-3 (Reverse)	5’-CATAGCTTGCTGTGCCATTT-3’
Rat IL-10 (Forward)	5’-CAGAGCTCAGGAAACTGCTG-3’
Rat IL-10 (Reverse)	5’-AGGCCTGGTCTTCTTTCAGA-3’
Rat Foxp3 (Forward)	5’-ACAAGGATCCTACCCACTGC-3’
Rat Foxp3 (Reverse)	5’-ATGCAGTTTAGCCCTTTGCT-3’
Rat PU.1 (Forward)	5’-AGACCAACCTATTAAGACCAT-3’
Rat PU.1 (Reverse)	5’-CATCCATCCAACCATTATCC-3’
Rat IRF4 (Forward)	5’-TGAAGTCGCTTATACATCTTATAG-3’
Rat IRF4 (Reverse)	5’-GTTGATACACTCTGGTAGGA-3’

Rat IL-9 (Forward)	5’- CCCTAAAGAGCAACAAGTGTCA -3’
Rat IL-9 (Reverse)	5’- GTGTTGCCTGCTGTGGTCT-3’

Suppl, Table 2: RT-PCR primers for human

Primer name	Sequences
hβ-actin (Forward)	5’-TGGACTTCGAGCAAGAGATG-3’
hβ-actin (Reverse)	5’-GAAGGAAGGCTGGAAGAGTG-3’
hMMP-3 (Forward)	5’-GCAAGACAGCAAGGCATAGA-3’
hMMP-3 (Reverse)	5’-TGGATAGGCTGAGCAAACTG-3’
